# Leonurine suppresses neuroinflammation through promoting oligodendrocyte maturation

**DOI:** 10.1111/jcmm.14053

**Published:** 2018-12-16

**Authors:** Min Jin, Qian Li, Yuting Gu, Bing Wan, Jiefang Huang, Xuanbai Xu, Rui Huang, Yanyun Zhang

**Affiliations:** ^1^ Medical College of Soochow University Soochow University Suzhou Jiangsu China; ^2^ Key Laboratory of Tissue Microenvironment and Tumor Shanghai Institutes for Biological Sciences Chinese Academy of Sciences Shanghai Jiao Tong University School of Medicine Shanghai China; ^3^ Department of Endocrinology and Metabolism Shanghai Jiaotong University Affiliated First People's Hospital Shanghai China

**Keywords:** CNS inflammation, EAE, leonurine, oligodendrocyte differentiation, remyelination

## Abstract

Focal inflammation and remyelination failure are major hallmarks of multiple sclerosis and its animal model, experimental autoimmune encephalomyelitis (EAE). In this study, we found that leonurine, a bioactive alkaloid, alleviated EAE disease severity along with reduced central nervous system inflammation and myelin damage. During the pathogenesis of EAE, leonurine dramatically suppressed the recruitment of encephalitogenic T cells into the central nervous system, whereas did not impair periphery immune responses and microglia activation. Mechanistically, leonurine protected mice against demyelination along with enhanced remyelination through promoting the maturation of oligodendrocytes in both EAE and cuprizone‐induced demyelination mouse models. Moreover, we identified that the expression of demethylase jumonji domain‐containing protein D3 was significantly enhanced upon treatment of leonurine, which suppressed the trimethylation of histone H3 lysine‐27 and enhanced oligodendrocyte maturation accordingly. Collectively, our study identified the therapeutic effect of leonurine on EAE model, which potentially represents a promising therapeutic strategy for multiple sclerosis, even other demyelination disorders.

## 1 INTRODUCTION

Multiple sclerosis (MS) is an autoimmune disease, characterized by central nervous system (CNS) inflammation, demyelination, axonal loss, and degeneration.^1^, ^2^ Myelin peptide MOG_35‐55_‐induced experimental autoimmune encephalomyelitis (EAE), which recapitulates CNS inflammation and demyelination, is a widely used autoimmune disease animal model for human MS.^3^, ^4^ Demyelination in EAE is mediated by peripheral preactivated, autoreactive, and myelin‐specific T cells that migrate and infiltrate into the CNS, where they become reactivated by antigen‐presenting cells and produce inflammatory cytokines, such as interleukins (e.g. IL‐1β, IL‐6, and IL‐17), tumour necrosis factor‐α, and interferon‐γ (IFN‐γ). These pathological events culminate in disseminated CNS inflammation, leading to the destruction of oligodendrocytes (OLs) and neurons, and then contributing to the damage of the myelin and axons.^3^, ^4^ Pharmacological strategies to modulate CNS inflammation can enable myelin repair and prevent subsequent neurodegeneration in this disease. Pathological studies also demonstrated that the endogenous myelination capacity decreases in MS patients.^5^, ^6^, ^7^ Boosting OL differentiation‐mediated remyelination to replenish damaged myelin is gaining more attention in MS therapeutics discovery.^8^, ^9^ In healthy brains, myelin is continually generated and dynamically remodelled by OLs, which are differentiated from oligodendrocyte progenitor cells (OPCs).^10^, ^11^, ^12^ During remyelination, OPCs migrate to the demyelinated lesion site, and then differentiates into OL and regenerates demyelinated myelin, protecting demyelinated axons from immune attack and irreversible degeneration.^13^ However, no drugs targeting remyelination has been reported until now.^9^ Exploring strategies to protect OLs and myelin during neuroinflammation is critical for MS therapy.

Leonurine (4‐guanidino‐n‐butyl syringate), a kind of bioactive alkaloid extracted from *Herba leonuri*,^14^ has been shown to exhibit anti‐inflammatory function. For example, Song et al showed that leonurine inhibited cyclooxygenase‐2 expression in lipopolysaccharide‐induced mouse mastitis.^15^ Li et al demonstrated that leonurine attenuated fibroblast‐like synoviocyte‐mediated synovial inflammation and joint destruction in rheumatoid arthritis.^16^ More recently, leonurine reportedly could decrease microglia or macrophage overactivation in Alzheimer's disease and monosodium urate crystal‐induced inflammation.^17^, ^18^ Besides, leonurine was surprisingly found exhibiting neuroprotective function in neurodegenerative diseases, such as Parkinson's disease,^19^ stroke,^20^, ^21^ and Alzheimer's disease,^17^ protecting brain from ischaemic injury and reducing neuron loss. In view of these findings, we set out to examine a possible role of leonurine in the CNS autoimmune disease EAE.

In this study, we firstly explored the therapeutic effect of leonurine on EAE mice, and found that leonurine alleviated disease severity of EAE reducing CNS inflammation and myelin damage. Leonurine reduced recruitment of encephalitogenic T cells into the CNS through suppressing CNS chemokine expression, whereas did not impair peripheral immune responses and microglia/macrophage activation. Interestingly, leonurine could protect mice from demyelination and enhance remyelination process, which was further validated in cuprizone‐induced demyelination mice and may confer leonurine‐ameliorated CNS inflammation in EAE model. This effect of leonurine on myelination was exerted by promoting OL differentiation partially through histone H3 lysine‐27 (H3K27) demethylase jumonji domain‐containing protein D3 (JMJD3). Therefore, our study demonstrates leonurine as a potential therapeutic compound for CNS autoimmune diseases.

## 2 MATERIALS AND METHODS

### 2.1 Induction and treatment of EAE model

Eight‐week‐old C57BL/6 male mice were purchased from the Shanghai Laboratory Animal Center of the Chinese Academy of Sciences, and kept under specific pathogen‐free conditions in the animal centre of Institute of Health Sciences, Chinese Academy of Sciences. Age‐matched mice were subcutaneously immunized with peptide MOG_35‐55_ (GL Biochem, Shanghai, China) mixed 1:1 with complete Freund's adjuvant (Sigma, St. Louis, MO, USA), followed by pertussis toxin (200 ng; List Biological Laboratories, Campbell, CA, USA) administration i.v. on day 0 and day 2, as previously described.^22^ Leonurine is chemically synthesized (Shifeng Biological Technology Company, Shanghai, China), and the purity is above 98%. For therapeutic protocol of EAE mice, leonurine (60 mg/kg) or vehicle (dimethyl sulphoxide) was daily administered (i.p.) from day 11 post immunization. Adoptive transfer EAE model was established as previously described.^23^, ^24^ Briefly, draining lymph node (DLN; proper axillary and accessory axillary) cells^25^ and splenocytes isolated from vehicle‐ or leonurine‐treated EAE mice on day 15 post immunization were re‐challenged with the MOG_35‐55_ (10 mg/mL) peptide for 3 days. 5 × 10^6^ viable cells were then transferred into sublethally irradiated mice (6 Gy). The recipients received pertussis toxin immediately after cell transfer and 2 days later. Mice were examined daily and scored for disease severity by the standard scale: 0, no clinical signs; 1, limp tail; 2, parapraxis (weakness, incomplete paralysis of one or two hind limbs); 3, paraplegia (complete paralysis of two hind limbs); 4, paraplegia with fore limb weakness or paralysis; 5, moribund or death. All animal procedures were approved by the Institutional Review Board of the Institute of Health Sciences, Shanghai Institutes for Biological Sciences, Chinese Academy of Sciences.

### 2.2 Histological analysis

Vehicle‐ and leonurine‐treated EAE mice were killed on day 28 post immunization. After cardiac perfusion with 4% paraformaldehyde, spinal cords were isolated, dehydrated with alcohol and embedded in paraffin. Spinal cord sections were then stained with haematoxylin and eosin (H&E) or luxol fast blue (LFB). Inflammation and demyelination scores were valued as previously described.^26^

### 2.3 Cell population analysis by flow cytometry

Spinal cord, brain, and DLN were isolated from vehicle‐ and leonurine‐treated EAE mice on day 19 post immunization. Mononuclear cells (MNCs) were isolated from CNS (a mix of spinal cord and brain) of by 37%/70% Percoll (GE Healthcare, Chicago, IL, USA) centrifugation or from DLN with Ficoll (STEMCELL Technologies, Vancouver, BC, Canada) centrifugation. MNCs were incubated with antibodies to CD4, CD8, CXCR3, or CCR5. To detect the pathogenic IFN‐γ‐ and IL‐17‐producing CD4^+^ T cell populations, MNCs were stimulated with phorbol‐12‐myristate‐13‐acetate (50 ng/mL; Sigma) and ionomycin (500 ng/mL; Sigma) in the presence of Brefeldin A (BD Bioscience, San Diego, CA, USA) for 5 hours. After that, the cells were incubated with antibody to CD4 and then antibodies to IFN‐γ and IL‐17α. The detection of regulatory T (Treg) cell was carried out using the mouse Treg cell staining kit according to the manufacturer's instructions. Stained cells were analysed by flow cytometry on BD FACS Callibur (BD Bioscience). These used antibodies were from eBioscience (San Diego, CA, USA).

### 2.4 Cell proliferation and measurement of cytokines

Reactive response of MNCs to MOG_35‐55_ stimulation was detected as previously described.^23^, ^24^ Briefly, MNCs were derived from spleen and DLN of vehicle‐ and leonurine‐treated EAE mice on day 15 post immunization and stimulated with MOG_35‐55_ (5 or 10 μg/mL) for 72 hours. One μC of [^3^H] thymidine (PerkinElmer, Waltham, MA, USA) was added into each well 16 hours before the end of the coculture. [^3^H] Thymidine incorporation was measured by MicroBeta TriLux Liquid Scintillation Counter (PerkinElmer). For cytokine measurements, supernatants were collected from cell cultures and diluted for the measurements of IFN‐γ, IL‐4, IL‐10, and IL‐17 by ELISA (R&D Systems, Minneapolis, MN, USA) according to the manufacturer's instructions.

### 2.5 Real‐time PCR

Total RNA was extracted with TRIzol (Life Technologies GmbH) and reverse‐transcribed into cDNA with the reverse transcription kit from TaKaRa (Tokyo, Japan). mRNA levels were measured by real‐time PCR with SYBR Green reagent (Roche, Natley, NJ, USA) and normalized to the *Actb* mRNA level on ABI Prism^®^ 7900HT Sequence Detection System (Applied Biosystems). The used primer sequences were listed in Table 1.

**Table 1:** Specific primers used in real‐time PCR analysis

Gene	Primer	Sequence (5′→3′)
*Actb*	F	CCACGAGCGGTTCCGATG
	R	GCCACAGGATTCCATACCCA
*Icam1*	F	CAATTTCTCATGCCGCACAG
	R	AGCTGGAAGATCGAAAGTCCG
*Vcam1*	F	TGAACCCAAACAGAGGCAGAGT
	R	GGTATCCCATCACTTGAGCAGG
*Ccl2*	F	ATTGGGATCATCTTGCTGGT
	R	CCTGCTGTTCACAGTTGCC
*Ccl3*	F	ACCATGACACTCTGCAACCA
	R	GTGGAATCTTCCGGCTGTAG
*Ccl5*	F	GCTGCTTTGCCTACCTCTCC
	R	TCGAGTGACAAACACGACTGC
*Cxcl10*	F	CCTATGGCCCTCATTCTCAC
	R	CTCATCCTGCTGGGTCTGAG
*Ccl20*	F	GGAAGGAAGAGGCGTCTGTA
	R	ACTCCTGGAGCTGAGAATGG
*Tnfa*	F	GGTCTGGGCCATAGAACTGA
	R	CAGCCTCTTCTCATTCCTGC
*Il1b*	F	GGTCAAAGGTTTGGAAGCAG
	R	TGTGAAATGCCACCTTTTGA
*Il6*	F	CCACGGCCTTCCCTACTTC
	R	CATTTCCACGATTTCCCAGA
*Il12b*	F	TGGTTTGCCATCGTTTTGCTG
	R	ACAGGTGAGGTTCACTGTTTCT
*Il23a*	F	AGCGGGACATATGAATCTACTAAGAGA
	R	GTCCTAGTAGGGAGGTGTGAAGTTG
*Mbp*	F	AGCCCTCTGCCCTCTCAT
	R	GGTAGTTCTCGTGTGTGAGTCCT‐
*Plp*	F	ATGGGCTTGTTAGAGTGTTGTG
	R	GTACCAGTGAGAGCTTCATGTC
*Ng2*	F	GCTGTCTGTTGACGGAGTGTT
	R	CGGCTGATTCCCTTCAGGTAAG
*Cc1*	F	CTTGTGGCCCAGTTAAAATCTGA
	R	CGCTTTTGAGGGTTGATTCCT

### 2.6 Primary microglia and cell line culture

Primary microglia cultures were prepared as previously described.^23^, ^24^ Briefly, cerebral cortical cells from newborn C57BL/6 mice were isolated after a 30‐minutes trypsinization (0.25%) and plated in 75‐cm^2^ culture flasks in DMEM with 10% heat‐inactivated foetal calf serum, 100 U/mL penicillin, and 100 mg/mL streptomycin (all from Life Technologies GmbH). The culture medium was changed after 24 hours and then once in every 4 days. Two weeks later, microglia were obtained by mild trypsinization. Purified microglia comprised a cell population in which 95% stained positively with CD11b antibodies. The murine macrophage cell line RAW 264.7 cells were purchased from ATCC (Manassas, VA, USA). Microglia and RAW 264.7 cells were activated by 100 ng/mL IFN‐γ for 24 hours (PeproTech, Rocky Hill, CT, USA).

### 2.7 Immunofluorescence

After cardiac perfusion with 4% paraformaldehyde, spinal cords or corpus callosum of mice were isolated, dehydrated with alcohol and embedded in paraffin. Spinal cord or corpus callosum sections were stained for primary antibodies including anti‐myelin basic protein (MBP), anti‐neural/glial antigen 2 (NG2; Abcam, Cambridge, MA, USA), and anti‐adenomatous polyposis coli (clone CC1; Millipore, Darmstadt, Germany). The secondary antibody was Alexa 488‐conjugated (Invitrogen). Nuclei were counterstained with DAPI (1 mg/mL; Sigma). The slides were mounted and visualized and captured by fluorescent microscope (Nikon, Tokyo, Japan).

### 2.8 Cuprizone‐induced demyelination mouse model

Eight‐week‐old female C57BL/6 mice were fed with 0.2% (w/w) cuprizone (Sigma) mixed into standard rodent chow.^27^, ^28^, ^29^ Three weeks later, the mice were given standard chow for 2 more weeks, and then daily injected (i.p.) with vehicle or leonurine (60 mg/kg). After 3 weeks treatment, the brain sections were prepared, and stained with LFB or immunofluorescence (IF) stained with NG2 and CC1. Quantitative analysis of myelination by LFB staining of corpus callosum in the brain of cuprizone‐induced demyelinated mice was carried out using Image‐Pro Plus (NIH, Bethesda, MD, USA) as previously reported.^30^ First, LFB staining images of corpus callosum were converted to a 256‐shade grey scale. Second, the 256 shades of grey were divided into 5 bins of 50 shades each: 0‐50, 51‐100, 101‐150, 151‐200, and 201‐256, with 0 being the pixel with darkest shade of grey and 256 being the pixel with the lightest shade of grey. Each bin was assigned an arbitrary colour: 0‐50 (red), 51‐100 (yellow), 101‐150 (green), 151‐200 (light blue), and 201‐256 (dark blue). Thus, each pixel was classified into one of the 5 bins based on the intensity of staining. Third, the number of objects in the corpus callosum region classified into each bin was counted.

### 2.9 Murine OPC preparation and OL differentiation

Oligodendrocyte progenitor cells used in this study were differentiated from murine cortical neural stem cells (NSCs), which were isolated as described previously^31^ with modification. Briefly, murine cortical NSCs were prepared from the brain on embryonic day 14.5 and cultured in serum‐free media (SFM) supplemented with B27 without vitamin A (Gibco), epidermal growth factor (20 ng/mL), and basic fibroblast growth factor (20 ng/mL; Peprotech). SFM contained DMEM/F12 (Gibco), glucose (30%), hepes buffer (1 mol/L), progesterone (2 mmol/L), putrescine (0.1 mmol/L), insulin‐transferrin‐selenium, and heparin (1.83 mg/mL). For OPC generation, the neurospheres were dissociated into single NSC by accutase (eBioscience), and then plated on a poly‐d‐lysine and laminin‐coated culture dish in SFM supplemented with B27 without vitamin A, basic fibroblast growth factor and platelet derived growth factor‐AA (40 ng/mL; Peprotech) for 3 days. To induce OL differentiation from OPCs in vitro, the culture medium was switched to SFM supplemented with B27 without vitamin A in the presence of triiodothyronine (T3; TCI Shanghai, Shanghai, China). Proliferation of differentiated OLs was analysed by bromodeoxyuridine (BrdU; Cell Signaling Technology, Danvers, MA, USA) assay according to the manufacturer's instructions.

### 2.10 Immunoblotting

Cells were harvested and lysed in the RIPA buffer (Beyotime, Haimen, China) containing phenylmethylsulfonyl fluoride protease inhibitor (Beyotime) for 30 minutes on ice. Lysates were clarified by centrifugation at 15 000 *g* for 30 minutes. Protein concentration of the supernatant fraction was determined by the Bradford assay (Thermo Fisher Scientific, NH, USA). Protein samples were diluted in 4× SDS loading buffer (TaKaRa) and heated to 95°C for 5 minutes and fractionated in a 10% or 8% SDS‐polyacrylamide gel. Proteins were electroblotted onto a polyvinylidene fluoride and incubated for 1 hour in 5% bovine serum albumin in phosphate buffer solution dissolved in phosphate buffer solution containing 0.1% Tween‐20 (PBST) at room temperature. The blotting membranes were incubated with primary antibodies to MBP (Abcam), trimethylated H3K27 (H3K27me3), H3K9me3, Histone 3, enhancer of zeste homolog 2 (EZH2), JMJD3 and β‐actin (Cell Signaling Technology) overnight at 4°C, extensively washed in PBST, incubated with HRP‐conjugated secondary antibody (Cell Signaling Technology) for 1 hour at room temperature, and washed again with PBST. The blotting membranes were developed with chemiluminescent reagents (Millipore, Billerica, MA, USA) according to the manufacturer's instructions. The densitometry of the bands was quantified using ImageJ software. OPCs cultured in OL differentiation medium were treated with vehicle or leonurine (5 μmol/L) in the presence of T3 for 5 days, combined with the EZH2 enzymatic inhibitor GSK126 or JMJD3 specific inhibitor GSK‐J4 at different concentrations. MBP expression was detected by immunoblotting analysis.

### 2.11 Ex vivo cerebellar slice culture

Cerebellar slices were isolated and cultured according to the protocol previously described.^32^ Briefly, whole cerebellum was collected at postnatal day (PD) 7 and cut into 300 μm sagittal slices on a microtome (Leica, Wetzlar, Germany). Slices were cultured in a DMEM/F12 supplemented with 15% heat‐inactivated horse serum (Gibco), B27 without vitamin A and platelet‐derived growth factor‐AA. After 1 day, the culture medium changed into DMEM/F12 with 15% heat‐inactivated horse serum and B27 without vitamin A. Cerebellar slices were then treated with leonurine (5 μmol/L) or vehicle for 1 or 2 days. The culture medium was changed daily and slices were lysed for immunoblotting.

### 2.12 Lentiviral vector construction

Oligonucleotides with the listed nucleotide sequences in Table 2 were used for the cloning of shRNA‐encoding sequences into a lentiviral vector PLKO.1 puro, a gift from Bob Weinberg (Addgene, Cambridge, MA, USA). High titre lentiviral stocks were produced, and murine NSCs were infected with scrambled control lentivirus (shNC) or lentivirus‐expressing shRNA inhibiting JMJD3 (sh*Jmjd3*‐1 and sh*Jmjd3*‐2) according to the manufacturer's protocol (http://www.addgene.org/tools/protocols/plko/). Cells resistant to puromycin (2 μg/mL) were selected for further study.

**Table 2:** shRNA sequence of JMJD3

Gene	Sequence (5′→3′)
shNC	CCGGCCTAAGGTTAAGTCGCCCTCGCTCGAGCGAGGGCGACTTAACCTTAGGTTTTTG
*Jmjd3*‐1	CCGGCCTGTTCGTTACAAGTGAGAACTCGAGTTCTCACTTGTAACGAACAGGTTTTTG
*Jmjd3*‐2	CCGGCCTCGTCATCTCAGTTCTCTACTCGAGTAGAGAACTGAGATGACGAGGTTTTTG

### 2.13 Statistical analysis

All measurement data are presented as mean ± SEM. SPSS software, version 20 (IBM, Armonk, NY, USA) was used for all statistical analyses. Significant differences were evaluated using an independent‐samples *t* test or Wilcoxon rank test. One‐way ANOVA was used to determine multigroup differences, and then we adopted the Bonferroni's or Dunn's Multiple Comparison Test for further analysis to determine the difference between two groups. Significance was expressed as: *P < 0.05, and **P < 0.01.

## 3 RESULTS

### 3.1 Leonurine ameliorates clinical severity of EAE mice

**Figure 1:**
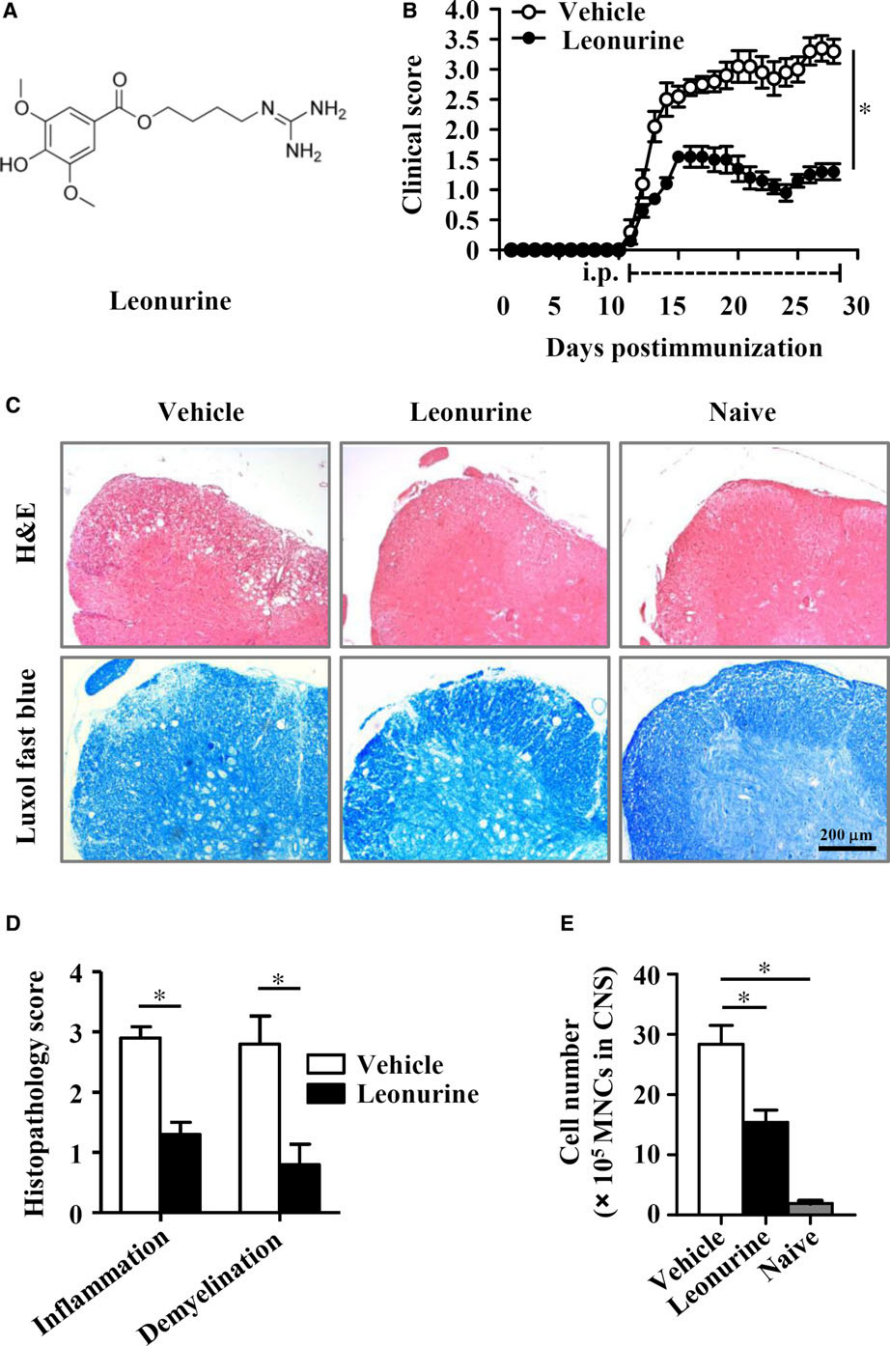
Leonurine alleviates CNS inflammation and myelin damage in EAE mice. (A) Chemical structure of leonurine. (B) Clinical scores of MOG_35‐55_‐induced EAE mice that were injected (i.p.) daily with leonurine (60 mg/kg) or vehicle from day 11 post immunization (n = 10). (C) Spinal cord sections from naive mice, vehicle‐treated EAE mice and leonurine‐treated EAE mice on day 28 post immunization were obtained and stained with H&E (top) or LFB (bottom). Scale bars, 200 μm. (D) Histopathology score of CNS inflammation and demyelination was quantified using H&E and LFB staining on day 28 post immunization (n = 5). (E) The absolute numbers of MNCs in the CNS from naive mice, vehicle‐treated EAE mice and leonurine‐treated EAE mice on day 19 post immunization were quantified (n = 5). Statistical significance indicated as *P < 0.05

To evaluate the potential effect of leonurine on MS, we tested the therapeutic effect of leonurine on MOG_35‐55_‐induced EAE mice. The molecular structure of leonurine was shown in Figure 1A. We treated the EAE mice with leonurine through injection (i.p.) from day 11 post immunization, which represented disease onset in EAE mice. The results revealed that leonurine treatment substantially alleviated the disease severity of EAE mice compared with vehicle treatment (Figure 1B). The observed therapeutic effects of leonurine were consistent with much less infiltration of inflammatory cells and fewer demyelinated plaques in the white matter of leonurine‐treated EAE mice compared with vehicle‐treated EAE mice (Figure 1C,D). Besides, the absolute numbers of MNCs in the CNS were also decreased after leonurine treatment (Figure 1E). These data suggested that leonurine alleviated disease severity of EAE, reducing CNS inflammation and myelin damage.

### 3.2 Leonurine prevents the recruitment of encephalitogenic T cells into the CNS

**Figure 2:**
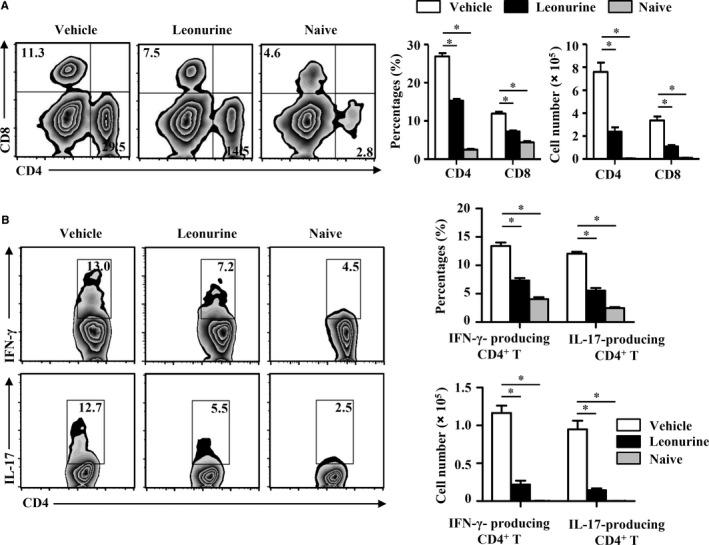
Leonurine prevents encephalitogenic T cell infiltration into the CNS in EAE mice. MNCs were isolated from the CNS of naive mice, vehicle‐treated EAE mice and leonurine‐treated EAE mice on day 19 post immunization. (A) Cells were analysed for expression of CD4 and CD8 in the lymphocyte gate by flow cytometry. The percentages and absolute numbers of CD4^+^ and CD8^+^ cells in the CNS were shown (n = 5). (B) The pathogenic IFN‐γ‐ and IL‐17‐producing CD4^+^ T cells were analysed by flow cytometry, and the percentages and absolute numbers in the CNS were shown (n = 5). Statistical significance indicated as *P < 0.05

The infiltrated autoreactive T cells are known required for the induction of CNS inflammation and pathology of EAE.^4^, ^33^ Consistent with the histological results, we found that the percentages and absolute numbers of CD4^+^ and CD8^+^ T cells in the CNS of EAE mice were dramatically decreased after leonurine treatment (Figure 2A). Accordingly, the infiltration of the pathogenic IFN‐γ‐ and IL‐17‐producing CD4^+^ T cells were also significantly decreased in the CNS of EAE mice after leonurine treatment (Figure 2B).

**Figure 3:**
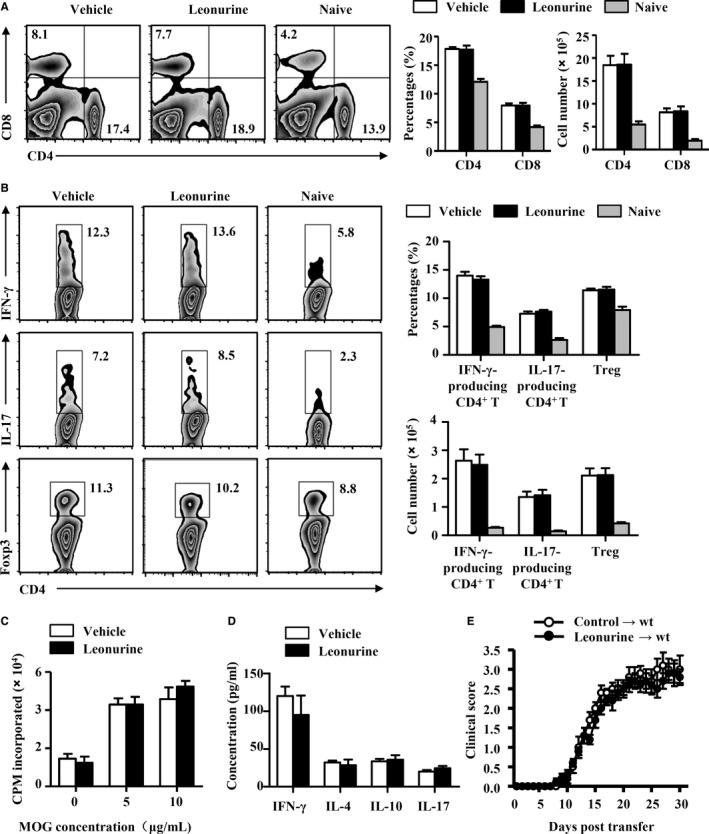
Leonurine does not impair immune responses in the periphery of EAE mice. MNCs were isolated from the DLN of naive mice, vehicle‐treated EAE mice and leonurine‐treated EAE mice on day 19 post immunization. (A) Cells were analysed for expression of CD4 and CD8 in the lymphocyte gate by flow cytometry. The percentages and absolute numbers of CD4^+^ and CD8^+^ cells in the DLN were shown (n = 5). (B) The pathogenic IFN‐γ‐ and IL‐17‐producing CD4^+^ T cells were analysed by flow cytometry, Treg cells were analysed for expression of Foxp3 in the CD4^+^ gate by flow cytometry, and the percentages and absolute numbers in the DLN were shown (n = 5). (C, D) MNCs were isolated from the DLN and spleen of vehicle‐ and leonurine‐treated EAE mice on day 15 post immunization and then stimulated with MOG_35‐55_ (5, 10 μg/mL) for 72 h ex vivo. Cell proliferation was examined by [^3^H] thymidine incorporation, and the levels of cytokine IFN‐γ, IL‐4, IL‐10, and IL‐17 were analysed by ELISA (n = 4). (E) DLN cells and splenocytes from vehicle‐ and leonurine‐treated EAE mice on day 15 post immunization were transferred into sublethally irradiated mice. Mice were monitored and scored daily (n = 5). Statistical significance indicated as *P < 0.05

We next explored whether leonurine impaired encephalitogenic T cell response in the periphery. Unexpectedly, the percentages and absolute number of CD4^+^ and CD8^+^ T cells were similar in the DLN of vehicle‐ and leonurine‐treated EAE mice (Figure 3A). There were also no considerable changes of IFN‐γ‐ and IL‐17‐producing CD4^+^ T cells and Treg cells in the DLN of EAE mice after leonurine treatment (Figure 3B). In addition, leonurine treatment had no obvious effects on the proliferation and cytokine production of the encephalitogenic T cells that re‐stimulated with MOG peptides in vitro compared with vehicle controls (Figure 3C,D). Accordingly, the recipient mice adoptively transferred with peripheral encephalitogenic T cells from vehicle‐ or leonurine‐treated EAE mice, developed similar typical EAE clinical symptoms (Figure 3E). These results suggested that the ameliorated EAE pathology conferred by leonurine was not caused by an impaired encephalitogenic T cell response in the periphery, but due to the inhibited infiltration of pathogenic autoimmune T cells into the CNS.

### 3.3 Leonurine does not affect the inflammatory response in microglia

**Figure 4:**
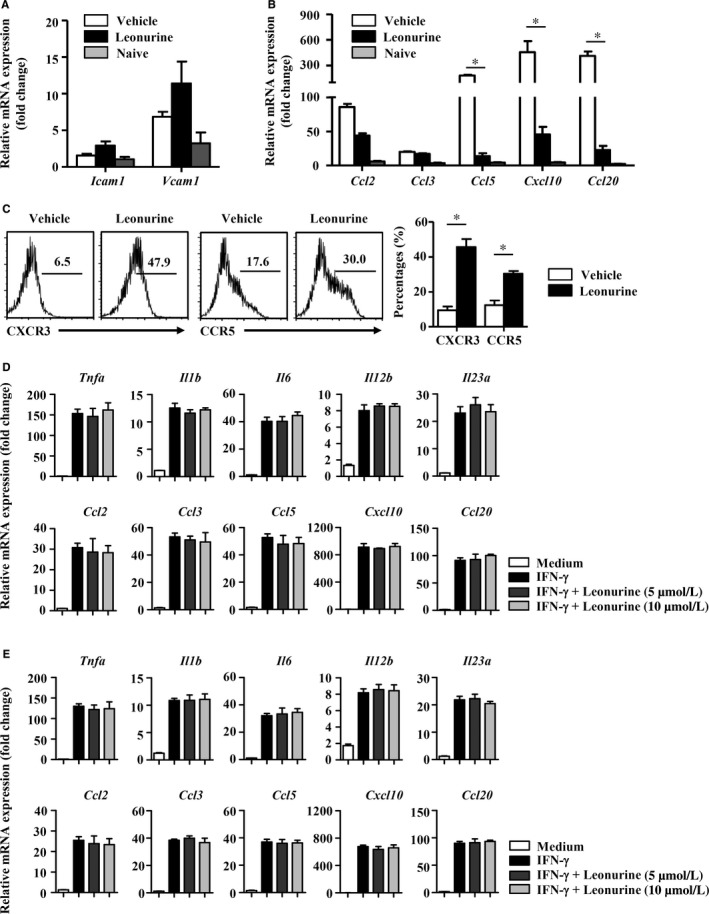
Leonurine does not affect the inflammatory response in microglia/macrophage. (A) Expression of *Icam1* and *Vcam1* in the CNS of naive mice, vehicle‐treated EAE mice and leonurine‐treated EAE mice on day 19 post immunization was measured by real‐time PCR (n = 4). (B) Expression of chemokine *Ccl2*,* Ccl3*,* Ccl5*,* Cxcl10*, and *Ccl20* in the CNS of naive mice, vehicle‐treated EAE mice and leonurine‐treated EAE mice on day 19 post immunization was measured by real‐time PCR (n = 4). (C) Flow cytometry analysis of the proportion of CXCR3^+^ CD4^+^ and CCR5^+^ CD4^+^ T cells in the DLN of vehicle‐ and leonurine‐treated EAE mice on day 19 post immunization. The percentages and absolute numbers of CXCR3^+^ CD4^+^ and CCR5^+^ CD4^+^ T cells in the DLN were shown (n = 4). (D) Primary microglia were isolated as described. Primary microglia were treated with vehicle or leonurine (5, 10 μmol/L) in the presence of IFN‐γ or not for 24 h. Expression of *Tnfa*,* Il1b*,* Il6*,* Il12b*,* Il23a*,* Ccl2*,* Ccl3*,* Ccl5*,* Cxcl10*, and *Ccl20* were measured by real‐time PCR (n = 4). (E) The murine macrophage cell line RAW 264.7 cells were treated with vehicle or leonurine (5, 10 μmol/L) in the presence of IFN‐γ or not for 24 h. Expression of *Tnfa*,* Il1b*,* Il6*,* Il12b*,* Il23a*,* Ccl2*,* Ccl3*,* Ccl5*,* Cxcl10*, and *Ccl20* were measured by real‐time PCR (n = 4). Statistical significance indicated as *P < 0.05

The recruitment of encephalitogenic T cells into CNS depends on the release of chemotactic mediators and the expression of cell adhesion molecules in the CNS.^34^, ^35^ Leonurine exhibited no significant effect on the expression of adhesion molecule *Icam1* and *Vcam1* in the CNS of EAE mice, whereas chemokine *Ccl2*,* Ccl3*,* Ccl5*,* Cxcl10*, and *Ccl20* were reduced significantly in the CNS of leonurine‐treated mice compared with vehicle‐treated mice (Figure 4A,B). Accordingly, the percentages and absolute numbers of CXCR3^+^/CCR5^+^CD4^+^ encephalitogenic T cells were significantly higher in the DLN of leonurine‐treated mice than vehicle‐treated mice (Figure 4C). Thus, leonurine seemed to function directly in CNS‐resident cells to suppress local neuroinflammation, and then inhibit the recruitment of autoimmune T cells into the CNS during EAE pathogenesis.

In CNS of EAE mice, activated microglia/macrophage produce proinflammatory factors that are toxic to the CNS and chemokines that promote inflammatory infiltration into the CNS.^3^, ^4^, ^36^ We therefore explored the effects of leonurine on proinflammatory activities of CNS‐resident cells such as microglia and infiltrated macrophage. To examine the modulatory effect of leonurine on microglia, primary cultured microglia were prepared and activated with IFN‐γ. The mRNA abundance for *Tnfa*,* Il1b*,* Il6*,* Il12b*,* Il23a*,* Ccl2*,* Ccl3*,* Ccl5*,* Cxcl10*, and *Ccl20* were significantly upregulated in IFN‐γ‐treated microglia. However, this upregulation was not affected by leonurine addition to microglia cultures (Figure 4D). Besides, leonurine also did not change the production of IFN‐γ‐induced proinflammatory cytokine and chemokines in the murine macrophage cell line RAW 264.7 (Figure 4E). Astrocytes have also been reported producing chemokines and playing a dominant role in the regulation of leucocyte recruitment.^37^, ^38^, ^39^, ^40^ Primary astrocytes were isolated, and our results showed that IFN‐γ‐up‐regulated mRNA expression of *Ccl2*,* Ccl3*,* Ccl5*,* Cxcl10*, and *Ccl20* was also not affected by leonurine addition to astrocytes cultures (Figure S1). These results collectively demonstrated that the suppressed EAE by leonurine was not due to the inhibition of microglia/macrophage inflammatory activation.

### 3.4 Leonurine promotes remyelination in EAE mice

**Figure 5:**
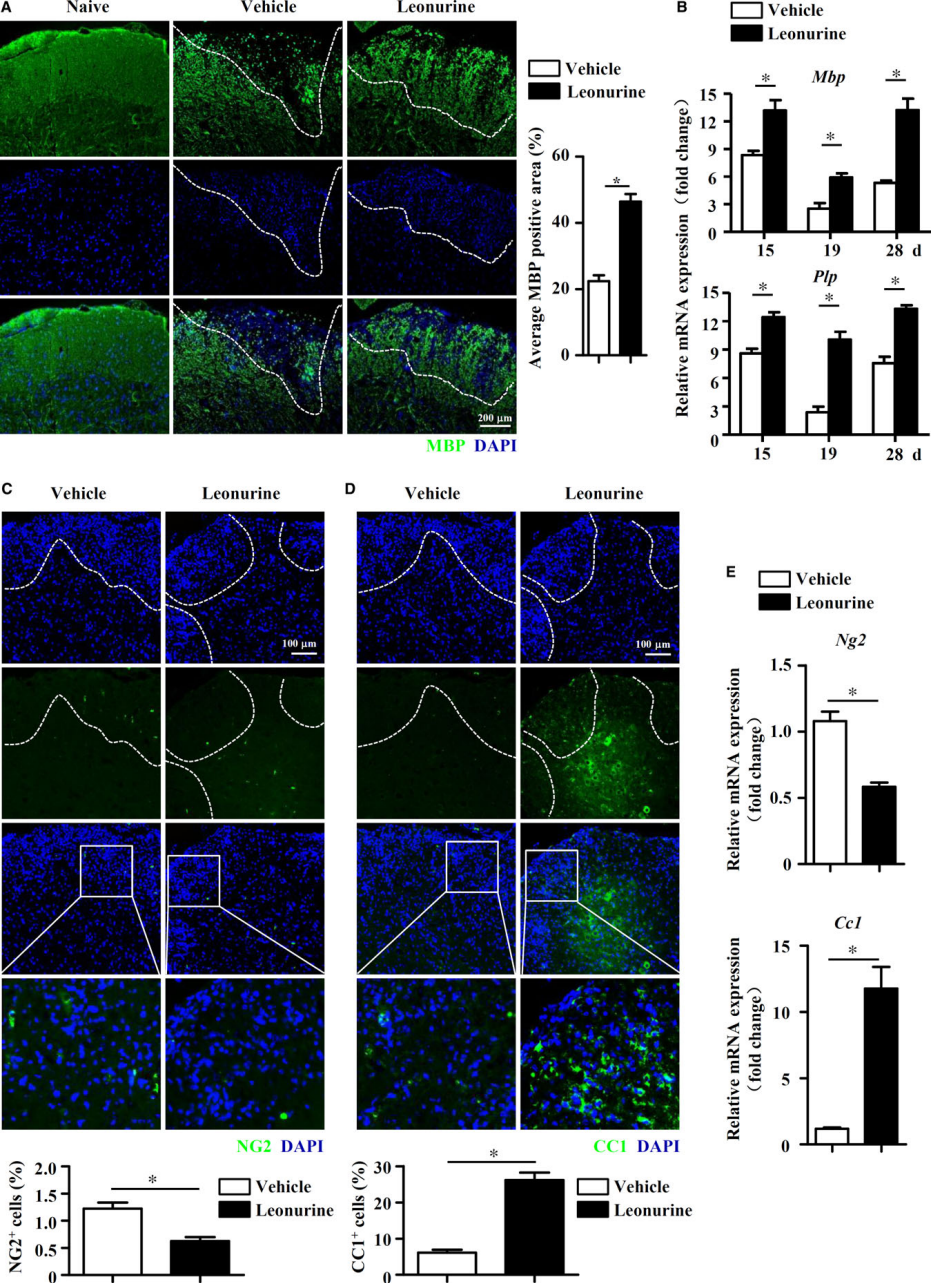
Leonurine promotes remyelination in EAE mice. (A) Spinal cord sections were excised from vehicle‐ and leonurine‐treated EAE mice on day 28 post immunization and analysed for MBP expression by IF staining. Quantification of MBP expression in the inflammatory loci was shown (n = 4). Scale bars, 200 μm. (B) Expression of *Mbp* and *Plp* in the spinal cords of vehicle‐ and leonurine‐treated EAE mice on day 15, 19, and 28 post immunization were measured by real‐time PCR (n = 4). (C, D) Spinal cord sections were excised from vehicle‐ and leonurine‐treated EAE mice on day 28 post immunization. Spinal cord sections were analysed for NG2 and CC1 expression by IF staining. Quantification of NG2 and CC1 expression in the inflammatory loci was shown (n = 4). Scale bars, 100 μm. (E) Expression of *Ng2* and *Cc1* in the spinal cords of vehicle‐ and leonurine‐treated EAE mice on day 28 post immunization were measured by real‐time PCR (n = 4). Statistical significance indicated as *P < 0.05

The amelioration of EAE pathology always results from two major aspects of function, the suppression of CNS inflammation and the promotion of remyelination,^41^ which promoted us to explore whether leonurine directly affected remyelination, and then conferred leonurine‐ameliorated CNS inflammation in EAE mice. We then analysed the expression of myelin proteins or genes, such as MBP and proteolipid protein (PLP), which were increased during remyelination and believed to reflect an important step leading to the remyelination process.^42^ As shown in Figure 5A, IF staining of spinal cords showed that nearly 2 folds of MBP expression was observed in the inflammatory loci of leonurine‐treated mice compared with vehicle‐treated mice. The mRNA levels of *Mbp* and *Plp* were also increased in the CNS after leonurine treatment (Figure 5B), indicating that leonurine indeed promoted the remyelination process in EAE mice.

Remyelination is a complicated process that OPCs differentiate into immature OLs and consequently mature OLs, which possesses the capability of myelination.^43^ Thus, we utilized stage‐specific markers, such as NG2 for OPCs and CC1 for newly generated OLs, to evaluate remyelination in the spinal cords of EAE mice on day 28 post immunization after leonurine treatment. Sporadic NG2^+^ OPCs were observed in the spinal cords of vehicle‐ and leonurine‐treated mice, and leonurine treatment reduced the number of NG2^+^ OPCs in the inflammatory loci (Figure 5C). However, there were much more CC1^+^ newly generated OLs in the inflammatory loci of the spinal cords of leonurine‐treated mice than that of vehicle‐treated mice (Figure 5D). The mRNA expression of *Ng2* and *Cc1* in the CNS displayed similar pattern (Figure 5E). These results implied that leonurine may promote the remyelination in the CNS of EAE mice through enhancing OPC differentiation into mature OLs.

### 3.5 Leonurine enhances remyelination in cuprizone‐induced demyelination model

**Figure 6:**
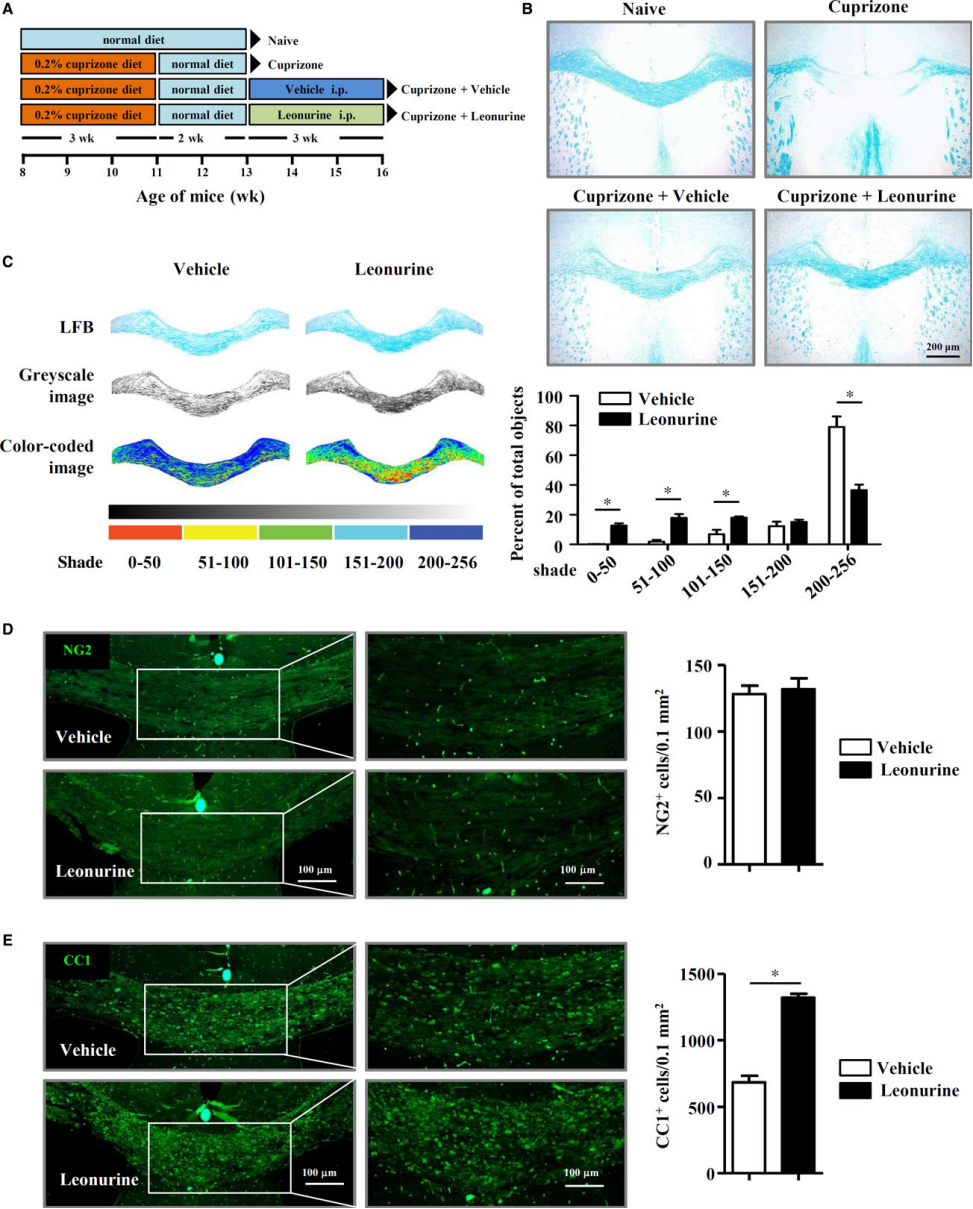
Leonurine enhances remyelination in cuprizone‐induced demyelination model. (A) Schematic protocol of cuprizone‐induced demyelination to identify the effect of leonurine on spontaneous remyelination. (B) Representative LFB staining images of the corpus callosum sections after cuprizone and leonurine treatment. (C) Representative LFB staining images of corpus callosum converted to a 256‐shade grey scale with or without arbitrary colours. Each bin with corresponding peseudo colour. Quantification of each bin in LFB staining images was shown. Leonurine treatment increased in the darker pixels (1‐50, 51‐100, and 101‐150) and reduced the number of lighter pixels (201‐256) (n = 4). (D, E) IF staining of NG2 and CC1 in the corpus callosum of mice after 3 weeks treatment of vehicle and leonurine. Quantification of the numbers of NG2^+^ cells and CC1^+^ cells per 0.1 mm^2^ in the corpus callosum was shown (n = 4). Scale bars, 100 μm. Statistical significance indicated as *P < 0.05

We next analysed the effects of leonurine on remyelination using cuprizone‐induced nonimmune‐mediated demyelination model, in which cuprizone induces the cell death of OLs while spares OPCs.^27^, ^28^, ^29^ After fed with a 0.2% (w/w) cuprizone diet for 3 weeks, mice were given standard chow for 2 more weeks. Significant demyelination was induced after 2‐week standard chow, and spontaneous remyelination occurred after that. Vehicle or leonurine was administered (i.p.) when spontaneous remyelination started (Figure 6A). Although spontaneous remyelination occurred in vehicle‐treated mice, remyelination in leonurine‐treated mice was significantly enhanced as compared with that of control mice (Figure 6B). Quantification of the remyelinated corpus callosum by grey scale analysis showed that leonurine treatment increased the number of darker pixels (1‐50, 51‐100, and 101‐150) and reduced the number of lighter pixels (201‐256) (Figure 6C), indicating that leonurine promoted spontaneous remyelination. The OL differentiation status was further quantified by NG2 and CC1 expression in the corpus callosum region. Although the number of NG2^+^ OPCs at the corpus callosum region displayed no obvious difference (Figure 6D), the number of CC1^+^ newly formed OLs increased significantly in the corpus callosum after leonurine treatment (Figure 6E), indicating that leonurine enhanced spontaneous remyelination in vivo by inducing OL differentiation.

### 3.6 Leonurine promotes OL differentiation through increasing JMJD3 in vitro

**Figure 7:**
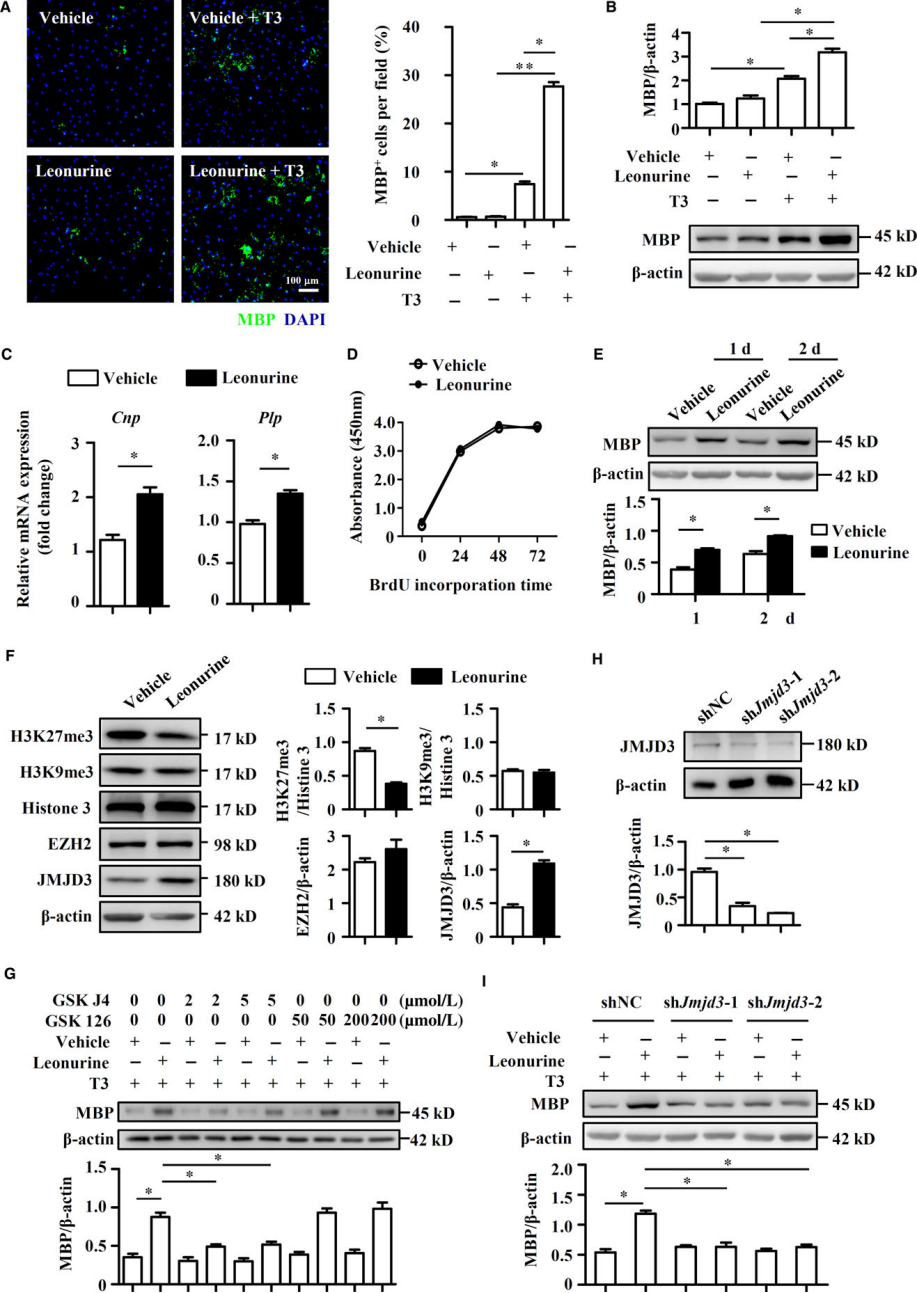
Leonurine augments in vitro OL differentiation through augmenting JMJD3 signaling. OL differentiation of OPCs derived from NSCs was induced as described in Section 2. (A, B) OPCs cultured in OL differentiation medium were treated with vehicle or leonurine (5 μmol/L) in the presence of T3 or not for 5 days. Cells were IF stained for MBP expression. Quantification of the percentages of MBP ^+^ cells per field was shown (n = 4). Scale bars, 100 μm. MBP protein expression was detected by immunoblotting analysis, and the densitometry of the bands were quantified using ImageJ software (n = 4). (C) OPCs cultured in OL differentiation medium were treated with vehicle or leonurine (5 μmol/L) in the presence of T3 for 5 days. Expression of *Cnp* and *Plp* was measured by real‐time PCR (n = 4). (D) BrdU analyses of proliferation of differentiated OLs treated with vehicle or leonurine (5 μmol/L) at the indicated times. (E) Cerebellar slices were isolated and cultured according to the protocol previously described. Cerebellar slices were then treated with leonurine (5 μmol/L) or vehicle for 1 or 2 days and slices were lysed for immunoblotting. The densitometry of the bands was quantified using ImageJ software (n = 4). (F) OPCs cultured in OL differentiation medium were treated with vehicle or leonurine (5 μmol/L) in the presence of T3 for 5 days. H3K27me3, H3K9me3, Histone3, EZH2, and JMJD3 expression were detected by immunoblotting analysis, and the densitometry of the bands were quantified using ImageJ software (n = 4). (G) OPCs cultured in OL differentiation medium were treated with vehicle or leonurine (5 μmol/L) in the presence of T3 for 5 days, combined with GSK‐J4 or GSK‐126. MBP expression was detected by immunoblotting analysis, and the densitometry of the bands were quantified using ImageJ software (n = 4). (H, I) JMJD3 expression in shNC, sh*Jmjd3*‐1, and sh*Jmjd3*‐2 NSCs was detected by immunoblotting analysis. Differentiated OLs were induced from shNC, sh*Jmjd3*‐1, and sh*Jmjd3*‐2 NSCs, and MBP expression was detected by immunoblotting analysis. The densitometry of the bands was quantified using ImageJ software (n = 4). Statistical significance indicated as *P < 0.05, **P < 0.01

To further confirm the prodifferentiation effect of leonurine on OLs, we analysed OL differentiation in vitro, in which OLs were differentiated from mouse embryonic NSC‐derived OPCs as described previously.^30^, ^31^ The isolated NSCs formed neurosphere (Figure S2A) and expressed NSC specific markers, such as *Olig2*,* Sox10*, and *Nestin* (Figure S2B). OPCs and OLs were generated from NSCs and validated by *Pdgfra*,* Mbp*, and *Plp* expression (Figure S2C). Although incapable of inducing OL differentiation alone, leonurine dramatically enhanced OL differentiation in the presence of T3, which is a potent OL differentiation inducer,^44^, ^45^ evidenced by elevated MBP expression (Figure 7A,B). The mRNA levels of *Cnp* and *Plp* specific for OLs were also dramatically elevated by leonurine in the presence of T3 (Figure 7C). However, BrdU incorporation assay showed that leonurine had no obvious effects on OL proliferation (Figure 7D). The observation that leonurine‐promoted OL differentiation raised the question that whether it also promoted further myelination. We therefore set up a postnatal myelination system by culturing cerebellar slices of postnatal mice with leonurine. Cerebellar slices were prepared from mice at PD 7, when widespread myelination is not complete,^32^ and treated ex vivo with leonurine or vehicle. The results showed that leonurine significantly boosted MBP expression (Figure 7E). Taken together, our data established leonurine as a critical positive regulator of the OL differentiation and myelination.

To dissect the molecular mechanism by which leonurine promotes OL differentiation, we investigated the effect of leonurine on the methylation alteration in histone 3, which is involved in OL differentiation.^46^, ^47^ As shown in Figure 7F, we found that leonurine treatment specifically inhibited H3K27me3, whereas had no effect on H3K9me3. The methyltransferase EZH2 and demethylase JMJD3 are well‐known to regulate the diversified methylation status of H3K27. Interestingly, leonurine treatment did not affect the expression of EZH2, but dramatically enhanced the expression of JMJD3. In addition, enzymatic inhibition of EZH2 by GSK126 during OL differentiation did not impede the enhancement effect of leonurine, whereas the JMJD3‐specific inhibitor GSK‐J4 strongly abrogated the enhanced effect of leonurine on OL differentiation (Figure 7G). To further validate the role of JMJD3 in leonurine function, JMJD3 was knocked down in NSCs using a lentivirus‐expressing shRNA specific to *Jmjd3* (named sh*Jmjd3*‐1 and sh*Jmjd3*‐2), and NSCs infected with a lentivirus‐expressing scrambled shRNA (named shNC) were used as controls (Figure 7H). As expected, JMJD3 knockdown also dampened the OL prodifferentiation effect of leonurine (Figure 7I). Thus, our data suggested that leonurine elevated the OL differentiation dependent on increased JMJD3 expression.

## 4 DISCUSSION

The therapy for EAE and MS always includes two major aspects: the suppression of CNS inflammation and the promotion of remyelination.^41^ Our previous studies have demonstrated two therapeutic compounds, baicalein and 18β‐glycyrrhetinic acid, reduced microglia‐mediated CNS inflammation and demyelination and then ameliorated EAE.^23^, ^24^ In this study, we provided several lines of evidence that leonurine alleviated disease severity of EAE by promoting OL differentiation, which conferred reduced encephalitogenic T cells infiltration in the CNS, thereby protecting mice from demyelination and enhancing remyelination process.

Demyelination induced by autoreactive T cell response has long been considered critical for MS and EAE model. Leonurine reportedly displayed anti‐inflammatory function.^15^, ^16^, ^17^, ^18^ However, we found that leonurine had no effect on T cell response in the periphery, indicating by similar components of CD4^+^ and CD8^+^ T cells in the DLN of vehicle‐ and leonurine‐treated EAE mice, as well as similar proliferation, cytokine profile, and ability to induce EAE of MOG_35‐55_ challenged T cells from vehicle‐ and leonurine‐treated EAE mice. Nevertheless, leonurine could inhibit T cell infiltration in the CNS. We further found that reduced T cell infiltration was associated with reduced chemokine in the CNS. These chemokines were mainly produced by CNS‐resident cells such as microglia and infiltrating immune cells, which also produced toxic proinflammatory factors to CNS. Hong and Liu et al showed that leonurine could decreased microglia or macrophage overactivation in β‐amyloid_1‐40_ (Aβ_1‐40_)‐induced cognitive impaired rats.^17^, ^18^ However, our data showed that leonurine did not affect the expression of proinflammatory cytokines and chemokines in microglia/macrophage in vitro. Besides, many studies also demonstrated that astrocytes produce chemokines and play a dominant role in the regulation of leucocyte recruitment.^37^, ^38^, ^39^, ^40^ We showed that leonurine did not affect the expression of *Ccl2*,* Ccl3*,* Ccl5*,* Cxcl10*, and *Ccl20* of primary astrocytes stimulated with IFN‐γ in vitro.

During the pathogenesis of EAE, promoted remyelination could suppress neuroinflammation and reduce the recruitment of peripheral immune cells into the CNS. During myelin regeneration, OPC migrates to the demyelinated lesion site, and then differentiates into OL and regenerates demyelinated myelin.^13^ In MS, myelin can dynamically be regenerated during the early stage, but the regeneration ability fails at the late stage.^7^ Dampened OL differentiation may be the underlying mechanism, as abundant OPCs exist in chronic lesions of MS patients.^7^, ^48^, ^49^ Our study here showed that leonurine‐promoted OPC differentiation into OLs in EAE mice and cuprizone‐induced demyelinated mice, evidenced by increased intensity of newly generated OL and mature OL in pathological lesions. Similar results were obtained in OPC differentiation into OLs in vitro. Remyelination in the CNS of EAE mice was always accompanied by reduced CNS inflammation. We also showed that leonurine did not impair encephalitogenic T cell response in the periphery, and the production of proinflammatory factors or chemokines of microglia and astrocytes was not affected by leonurine. These indicated that leonurine‐promoted OL differentiation and CNS remyelination of EAE mice may confer leonurine‐ameliorated CNS inflammation in EAE.

Genomic analysis reveals that many transcription factors and lineage related genes in OPCs are dynamically regulated and show different expression patterns upon differentiation, the process of which may involve diverse epigenetic modifications.^50^ Indeed, several epigenetic enzymes have been reported to be essential for OL commitment.^51^, ^52^ Sher et al found that EZH2 was expressed in NSCs, OPCs and immature OLs, and was essential for OPC differentiation into OLs.^46^, ^47^ EZH2 and H3K27me3 guaranteed OL differentiation by cancelling the repression on oligodendrocytic lineage determining genes.^46^, ^47^ In this study, we found that leonurine‐promoted OL differentiation at least partially through H3K27 demethylase JMJD3. The involvement of JMJD3 in OL differentiation was consistent with the study of Park et al that JMJD3 was required for neural differentiation of NSCs.^53^ As JMJD3 always functions via H3K27 demethylation at specific genomic regions, it is interesting to explore how JMJD3 affects OL differentiation under leonurine regulation.

In summary, our findings demonstrate that leonurine promotes OL differentiation to protect mice from demyelination and enhance remyelination accompanied with reduced T cell infiltration in the CNS, which potentially provides a promising therapeutic strategy for MS, and even other demyelination disorders.

## CONFLICT OF INTEREST

The authors declare no conflict of interests.
