# Supplementary material for: Leonurine suppresses neuroinflammation through promoting oligodendrocyte maturation
Source: J Cell Mol Med. 2018 Dec 16;23(2):1470–85. doi: 10.1111/jcmm.14053 (PMC6349161; doi:10.1111/jcmm.14053)
Supplement: Supplementary file 1 [file JCMM-23-1470-s001.docx]

**Supplementary materials and methods**

**Primary astrocyte isolation and culture:** Cerebral cortices from newborn C57BL/6 mice were dissected, carefully stripped of their meninges, digested with 0.25% trypsinization and DNAse I (1 mg/ml) for 15 min, and dispersed to single-cell level by passing through a cell strainer (70 μm). Cerebral cortical cell suspension was then plated in poly-L-Lysin (Sigma) pre-coated 75-cm^2^ cell culture flasks in DMEM with 10% heat-inactivated fetal calf serum, 100 U/ml penicillin, and 100 mg/ml streptomycin. The culture medium was changed every 4-5 days. After 7-10 days, cells reached confluence, and were used for the continued three or four passages. Astrocytes were activated by 100 ng/ml IFN-γ for 24 hours.

**Table S1. Specific primers used in reverse transcription PCR analysis in Supplemental figure.**

| Gene | Primer | Sequence (5*'*→3*'*) |
| --- | --- | --- |
| Actb | F | CCACGAGCGGTTCCGATG |
|  | R | GCCACAGGATTCCATACCCA |
| Olig2 | F | AGACCGAGCCAACACCAG |
|  | R | AAGCTCTCGAATGATCCTTCTTT |
| Sox10 | F | ACACCTTGGGACACGGTTTTC |
|  | R | TAGGTCTTGTTCCTCGGCCAT |
| *Nestin* | F | CCCTGAAGTCGAGGAGCTG |
|  | R | CTGCTGCACCTCTAAGCGA |
| Pdgfra | F | TCCATGCTAGACTCAGAAGTCA |
|  | R | TCCCGGTGGACACAATTTTTC |
| *Mbp* | F | AGCCCTCTGCCCTCTCAT |
|  | R | GGTAGTTCTCGTGTGTGAGTCCT- |
| *Plp* | F | ATGGGCTTGTTAGAGTGTTGTG |
|  | R | GTACCAGTGAGAGCTTCATGTC |

**Supplemental figure legend**

**Figure S1** Leonurine does not affect chemokines of primary astrocytes *in vitro*. Primary astrocytes were isolated as described in Supplemental Materials and methods. Primary astrocytes were treated with vehicle or leonurine (5, 10 μM) in the presence of IFN-γ or not for 24 hours. Expression of *Ccl2*, *Ccl3*, *Ccl5*, *Cxcl10* and *Ccl20* were measured by real-time PCR (n=4).

**Figure S2** OL differentiation of OPCs derived from NSCs. OL differentiation of OPCs derived from NSCs were induced as described in Materials and methods. (**a**) The neurosphere of isolated NSCs. (**b** and **c**) Specific genes of NSCs, OPCs and OLs were analyzed by reverse transcription PCR and agarose gel electrophoresis, including *Olig2*, *Sox10*, *Nestin*, *Pdgfra*, *Mbp* or *Plp*. The primers used are listed in Table S1.
