# Supplementary figures and images for: Leonurine suppresses neuroinflammation through promoting oligodendrocyte maturation
Source: J Cell Mol Med. 2018 Dec 16;23(2):1470–85. doi: 10.1111/jcmm.14053 (PMC6349161; doi:10.1111/jcmm.14053)

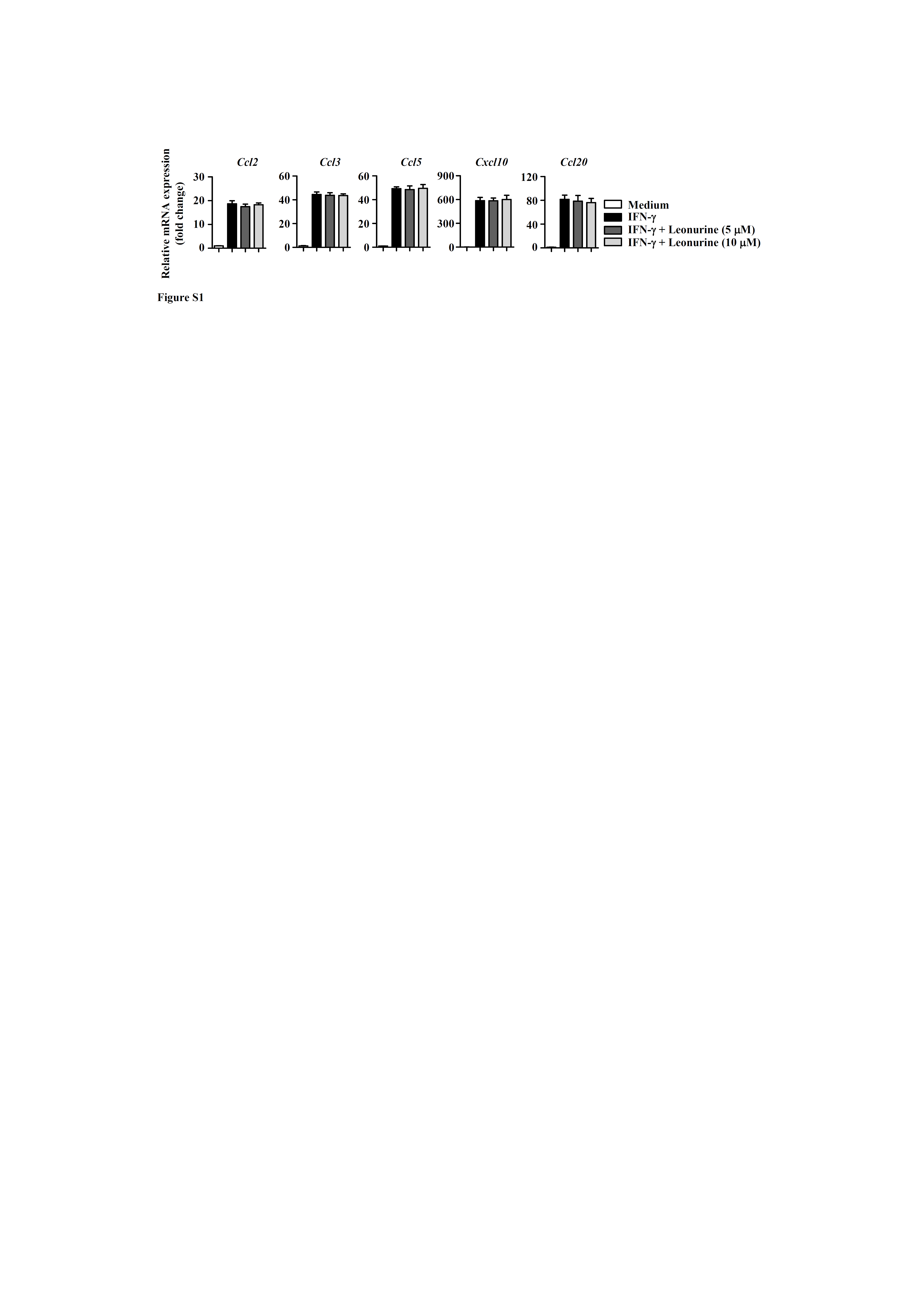

Supplement: Supplementary file 2 [file JCMM-23-1470-s002.tif]

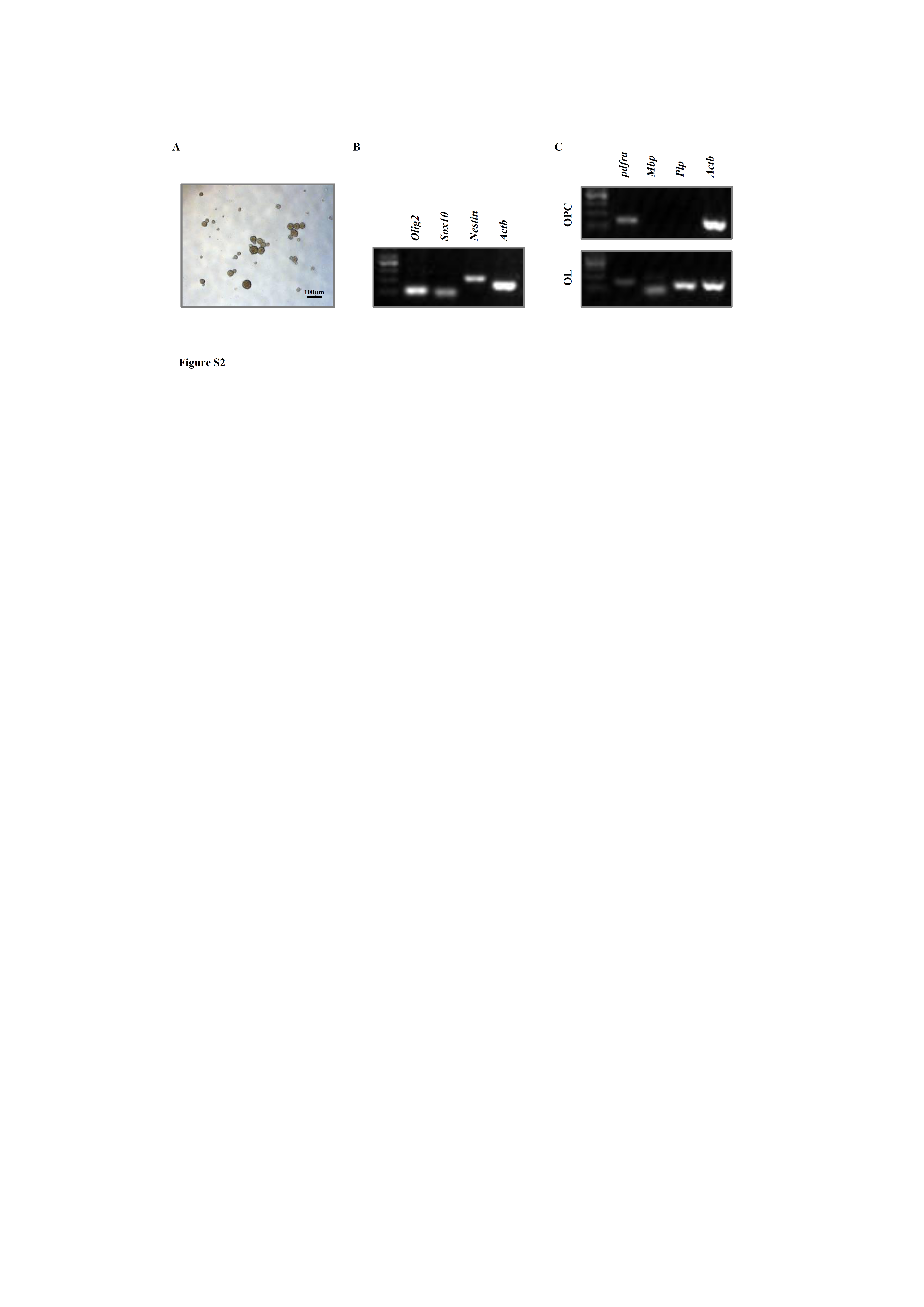

Supplement: Supplementary file 3 [file JCMM-23-1470-s003.tif]
